# Computational analysis of the hemodynamic characteristics under interaction influence of β-blocker and LVAD

**DOI:** 10.1186/s12938-018-0602-5

**Published:** 2018-12-03

**Authors:** Kaiyun Gu, Zhe Zhang, Yu Chang, Bin Gao, Feng Wan

**Affiliations:** 1 Peking University Third Hospital, Peking University Health Science Center, 49 North Garden Rd, Haidian District, Beijing, 100191 China; 2 College of Life Science & Bio-Engineering, Beijing University of Technology, Beijing, 100124 China

**Keywords:** Hemodynamics, Left ventricular assist devices, Multi-scale model, β-Adrenergic receptor antagonists

## Abstract

**Background:**

Hemodynamic characteristics of the interaction influence among support level and model of LVAD, and coupling β-blocker has not been reported.

**Methods:**

In this study, the effect of support level and model of LVAD on cardiovascular hemodynamic characteristics is investigated. In addition, the effect of β-blocker on unloading with LVAD is analyzed to elucidate the mechanism of LVAD coupling β-blocker. A multi-scale model from cell level to system level is proposed. Moreover, LVAD coupling β-blocker has been researching to explain the hemodynamics of cardiovascular system.

**Results:**

Myocardial force was decreased along with the increase of support level of LVAD, and co-pulse mode was the lowest among the three support modes. Additionally, the β-blocker combined with LVAD significantly reduced the left ventricular volume compared with LVAD support without β-blocker. However, the left ventricular pressure under both cases has no significant difference. External work of right ventricular was increased along with the growth of support level of only LVAD. The LVAD under co-pulse mode achieved the lowest right-ventricular EW among the three support modes.

**Conclusions:**

Co-pulse mode with β-blocker could be an optimal strategy for promoting cardiac structure and function recovery.

## Introduction

Left ventricular assist device (LVAD), as a novel gold standard plays a pivotal role of a bridge on heart transplantation, contributing to improving survival rate and being widely applied for patients with end-stage heart failure in clinics [1]. LVAD support results in a profound volume unloading in the left ventricle, causing dramatic reverse remodeling regarding structural, biochemical and genetic. Madigan et al. [2] reported reverse remodeling in terms of normalization of ventricle geometry, regression of hypertrophy, and normalized expression of excitation contraction genes (calcium adenosine triphosphatase 2α), indicating function recovery. Barbone et al. [3] found volume of cardiomyocytes decreased along with the reduced left ventricular pressure. Castagna et al. [4] reported low pulsatility reduced endothelial function due to lack of optimal cyclical stress. Rebholz et al. [5] showed that a pulsatile VAD stroke volume can be reduced by 71%, while maintaining an aortic pulse pressure of 30 mm Hg, avoiding suction events, reducing the ventricular stroke work and allowing the aortic valve to open. Canseco et al. [6] showed up to 60% decrease in mitochondrial content and 45% decrease in cardiomyocyte size and increase of cardiomyocyte proliferation in 10 patients with LVAD implantation. Despite the improved cardiac function after LVAD implantation, a gap still exists between reverse remodeling and complete functional recovery. Dandel et al. [7] reported that only 13% of non-ischemic patients of heart failure (HF) patient significantly improved in cardiac function and achieved the standard of explantation through 36-months follow-up investigation. Long-term LVAD support resulted in significant improvement in pulmonary artery pressure regardless of the pump generation. The improvement in hemodynamics observed during LVAD support was sustained 3–5 years posttransplant [56]. In addition, recurrence rate of cardiac function deterioration among patients with LVAD explantation is 52% in 1 year and 80% in 3 years.

Medicine is advancing toward the use of pharmaceuticals to enhance recovery in HF patients through LVAD and cellular mechanisms responsible for the improvement in left ventricular (LV) function is under extensive exploration. Soppa et al. [8] combined LVAD with pharmacological therapy which led to a substantially improved recovery rate in patients. Navaratnarajah et al. [9] suggested Ivabradine as a heart rate reduction agent that inhibit the pacemaker current in the sinoatrial node and enhance functional recovery in HF patients after receiving LVAD therapy. In addition, intracellular calcium concentration is critical for efficient myocardial force as a fatal parameter for the function recovery. Myocardial dysfunction is compensated for neuroendocrine release of norepinephrine and epinephrine, which stimulate β-adrenergic receptor [10]. Clinical treatment using β-adrenergic receptor antagonists (β-blocker) halted deterioration of cardiac function in end-stage HF patients by improving intracellular Ca^2+^ cycling to increase myocardial force [11]. Both LVAD and pharmacological therapy are beneficial for cardiac function. Therefore, a combination of optimized LVAD unloading with a standardized protocol of pharmacological therapy is worthy of being researched. Nevertheless, a comprehensive study on the effort support level, model of LVAD and LVAD coupling β-blocker on the hemodynamic characteristics has not been reported.

In the present study, we investigated the effect of support level and model of LVAD on cardiovascular hemodynamic characteristics, and the effect of β-blocker on unloading with LVAD Besides, we elucidate the mechanism of LVAD coupling β-blocker. A multi-scale model that cover Ca^2+^ transient, intracellular calcium concentration, cross-bridge dynamics, ventricle model and cardiovascular system were presented in this study. Blood assist index (BAI) (from 20 to 90%) and support models (constant speed, co-pulse, counter-pulse) were utilized to study the effect of LVAD on cardiovascular system. Here, β-blocker as a significant block β-adrenergic receptor has a role of adjusting the intracellular calcium concentration, meanwhile, has no effect of peripheral vascular, such as Bisoprolol and Metoprolol. The treatment effectiveness of LVAD coupling β-blocker was researched. Myocardial force, aortic pressure (AOP), left ventricular pressure (LVP), right ventricular pressure (RVP), arterial pressure (AP), aortic valve flow, pressure–volume loop (PV loop), external work (EW) and pulsatile ratio (PR) as the parameters have been applied to evaluating the state of cardiovascular system, ventricular unloading and the pulsatility of pressure.

## Materials and methods

**Fig. 1:**
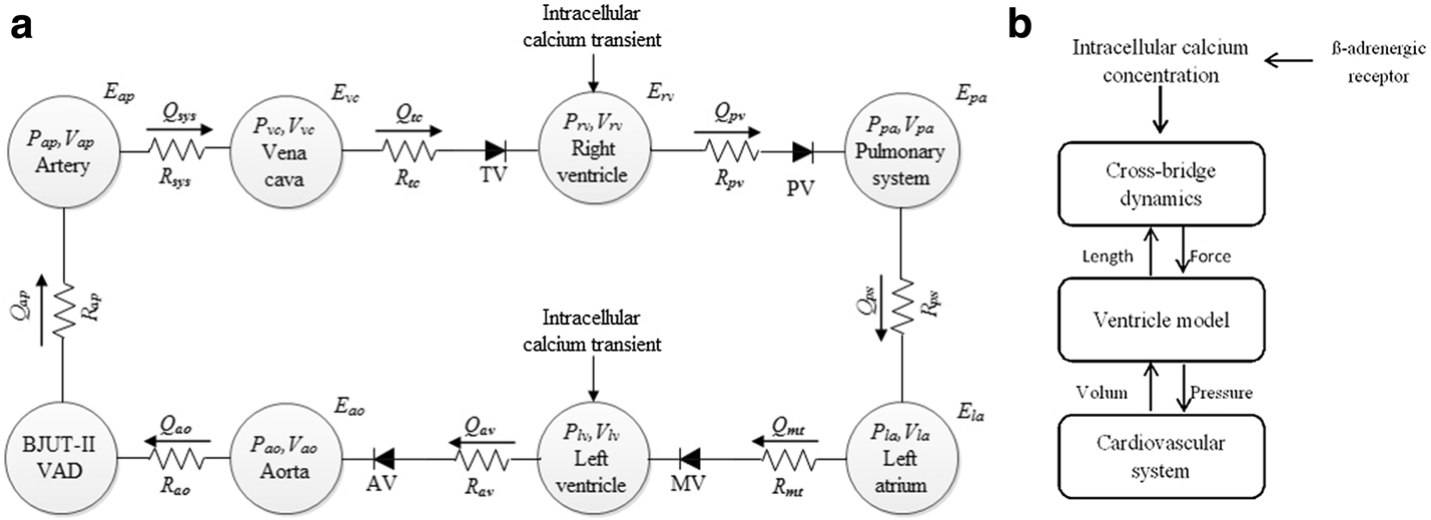
Schematic of multi-scale model.** a** Multi-scale lumped parameters model;** b** schematic of the interconnect model of the cardiovascular system

A multi-scale model from cell level to system level is created in Fig. 1a. This model consists of four components, calcium transient of human ventricular myocyte, the cross-bridge dynamics model, ventricle model and cardiovascular-pump system model (Fig. 1b). Based on multi-scale model, LVAD coupling β-blocker can be studied to explain the hemodynamic mechanism of cardiovascular system. Ca^2+^ released experimental model focuses on intracellular calcium ion concentration varies over time. Cross-bridge dynamics model focuses on binding Ca^2+^ to troponin and force generation depends on muscle length. Ventricle model is characterized by the pressure and the volume of cardiovascular system.

### Intracellular calcium transient

Intracellular calcium transient has been derived from Ref. [8]. According to previously published data [9], calcium transient is modified to reflect the variation of concentration in HF patients.

1 $$[{\rm Ca}^{2+ } ]_{lv} (t) = \left\{ {\begin{array}{ll} {\frac{{{\rm Ca}_{{max} ,lv} }}{2}\left( {1 - \cos \left( {\frac{\pi \cdot t}{{T_{1,lv} }}} \right)}\right) + 0.1}  & {\quad \; 0 {\text{ < t < }}T_{1,lv} } \\ {\frac{{{\rm Ca}_{{max} ,lv} }}{2}\left( {1 + \cos \left( {\frac{{\pi (t - T_{1,lv} )}}{{T_{2,lv} - T_{1,lv} }}} \right)}\right) + 0.1}  & {\quad \;T_{1,lv} < t < T_{2,lv} } \\ 0 & {\quad \,{\text{others}}} \\ \end{array} } \right.$$

where ${\rm Ca}_{{max} ,lv}$ is the maximum value of concentration. $T_{1,lv}$ is the time of peak calcium concentration, and $T_{2,lv}$ is the time when calcium concentration fell down to 0.1 μM [9]. The estimated intracellular calcium transient is shown in Fig. 1b and the parameter values are shown in Table 1.

**Table 1:** State variable

Parameter	Value	Unit
T_1.lv_	40.6	ms
T_2,lv_	130.2	ms
Ca_max,lv_	1.47	μM
Y_1_	39	μMs^−1^
Z_1_	30	s^−1^
Y_2_	1.3	s^−1^
Z_2_	1.3	s^−1^
Y_3_	30	s^−1^
Z_3_	1560	μMs^−1^
Y_4_	40	s^−1^
Y_d_	8	S μm^−2^
T_t_	70	μM
B	800	s^−1^
h_c_	0.005	μm
L_0_	1.17	μm

The chemical kinetics [12] are defined as follow:

2 $$\frac{d[T{\rm Ca}]}{dt} = Y_{1} \cdot [T] \cdot [{\rm Ca}^{2 + } ] + Z_{2} \cdot [T{\rm Ca}^{ * } ] - (Y_{2} \cdot e^{{ - R(L - L_{a} )^{2} }} + Z_{1} ) \cdot [T{\rm Ca}]$$

3 $$\frac{{d[T{\rm Ca}^{ * } ]}}{dt} = Y_{2} e^{{ - R(L - L_{a} )^{2} }} [T{\rm Ca}] + Z_{3} [T^{ * } ][{\rm Ca}^{2 + } ] - \left( {Z_{2} + Y_{d} \left( {\frac{dX}{dt}} \right)^{2} + Y_{3} } \right)\,[T{\rm Ca}^{ * } ]$$

4 $$\frac{{d[T^{ * } ]}}{dt} = Y_{3} [T{\rm Ca}^{ * } ] - \left( {Z_{3} [{\rm Ca}^{2 + } ] + Y_{4} + Y_{d} \left( {\frac{dX}{dt}} \right)^{2} } \right)[T^{*} ]$$

5 $$[T] = T_{t} - [T^{*} ] - [T{\rm Ca}] - [T{\rm Ca}^{*} ]$$

where $[{\rm Ca}^{2 + } ]$ is calcium concentration; $[T{\rm Ca}]$ presents the concentration of calcium bound to troponin; $[T{\rm Ca}]$ is concentration of troponin to bind myosin located on the thin filaments; $[T]$ is troponin concentration; $[T^{*}]$ is concentration of troponin bound to myosin; *X* is the difference of half-sarcomere length and cross-bridge length. Y_i_ (i = 1, 2, 3, 4) and Z_j_ (j = 1, 2, 3) are the coefficients of different directions. The parameter, *R,* function as the curvature of the function. *L* is a half-sarcomere of length. *La* is the overlap between thin and thick filaments. Overlap is maximal when *L *= *La*. These values have been referred from the literature [12].

### Cross-bridge dynamics model

The Negroni and Lascano model [12] was served as calculating the active force from sarcomeres.

6 $$\frac{dX}{dt} = B(L - X - h_{c} )$$

7 $$F_{a} = A\,\left( {[T{\rm Ca}^{*} ](t) + [T^{*} ](t)} \right)\left( {L(t) - X(t)} \right)$$

8 $$F_{p} = - K\,\left( {1 - \frac{L(t)}{{L_{0} }}} \right)$$

9 $$F(t) = F_{a} (t) + F_{p} (t)$$

where L represents half-sarcomere of length; X = L–h, h is the cross-bridge length; h_c_ is equilibrium value of the cross-bridge length; B is the rate when h reaches its equilibrium value h_c_; $F_{a}$ represents active force; $F_{p}$ represents passive forces; K is the stiffness of the element.

### Ventricle model

The chamber of heart is assumed as a hemispherical shape, and the muscle fiber is modeled as warp direction. Base on Laplace law, the relationship between stress and pressure of ventricular is defined as following:

10 $$P(t) = \frac{2F(t)h}{R(t)}$$

where $P(t)$ represents center of pressure; *h* represents myocardium thickness; $R(t)$ is the radius of ventricular, which can be used to calculate the volume of ventricular. In addition, the relationship between strain of myocardial fibers and radius of ventricular was defined as follow:

11 $$\frac{R(t)}{{R_{0} }} = \frac{L(t)}{{L_{0} }}$$

where $R_{0}$ is radius without stress, $L_{0}$ is myocardial fibers length without stress.

### Cardiovascular–pump system model

A lumped parameter model based on Smith et al. [13] and the mathematical model of BJUT-II VAD pump [14] were used to simulate the cardiovascular system with pump. This model consisted eight parts: left ventricular, right ventricular, aorta, artery, vena cava, pulmonary artery, pulmonary veins and BJUT-II VAD pump (Fig. 1a). In addition, four valves including aortic valve, tricuspid valve, pulmonary valve, mitral valve were described in this model. The active left ventricle in this model is presented above ventricle model.

The mathematic model of BJUT-II VAD [14] describes as a function of the flow rate, pressure head and rotational speed of the pump:

12 $$P_{p} (\omega ,Q_{PO} ) = 0.0115\omega^{2} + 0.079\omega - 15.5 - (0.086\omega - 0.58)Q_{PO} + L_{P} \frac{{dQ_{PO} }}{dt}$$

where $Q_{PO}$ is the flow rate of the pump (L/min). $P_{p}$ is the pressure head of the pump (mmHg). $\omega$ is the rotational speed (R/s). $\omega_{\text{limit}}$ denotes the threshold speed. $L_{P}$ is the inertia of blood in intra-aorta pump.

### Hemodynamic analysis

In this paper, EW and PR are employed to evaluate the hemodynamic effect. The EW of the ventricle is used to evaluate the declining cardiac performance in heart failure, which could be described as following:

13 $$EW = \frac{0.0022}{T}*\left( {\int_{0}^{T} {LVP(t)*CO(t)dt} } \right)$$

where $EW$ whose unit is watts reflects the ventricular external work. T is the cardiac cycle. $LVP(t)$ whose unit is mmHg represents the aortic pressure; $CO(t)$ whose unit is L/min is the cardiac output.

Regarding the previous research [15], the pulsatile ratio (PR) as an index to reflect the pulsatility of pressure was defined as follows:

14 $$PR = \frac{{AP_{{max} } - AP_{{min} } }}{MAP}$$

where $AP_{{max} }$ is the maximum value of the arterial pressure during the one cardiac cycle; $AP_{{min} }$ represents the minimum value of arterial pressure during the same cardiac cycle; MAP denotes the mean arterial pressure.

In addition, Blood assist index (BAI) [16], the ratio of the power of LVAD and total power of the cardiovascular system, is an indicator utilized to evaluate the support level, which reflects the energy distribution between LVAD and the native heart. In this study, constant speed, the widespread model in clinical, was used to research the effect of support levels of LVAD on cardiovascular hemodynamic. Meanwhile, BAI value was increased from 20 to 90% to reflect on the unloading situations.

## Results

**Fig. 2:**
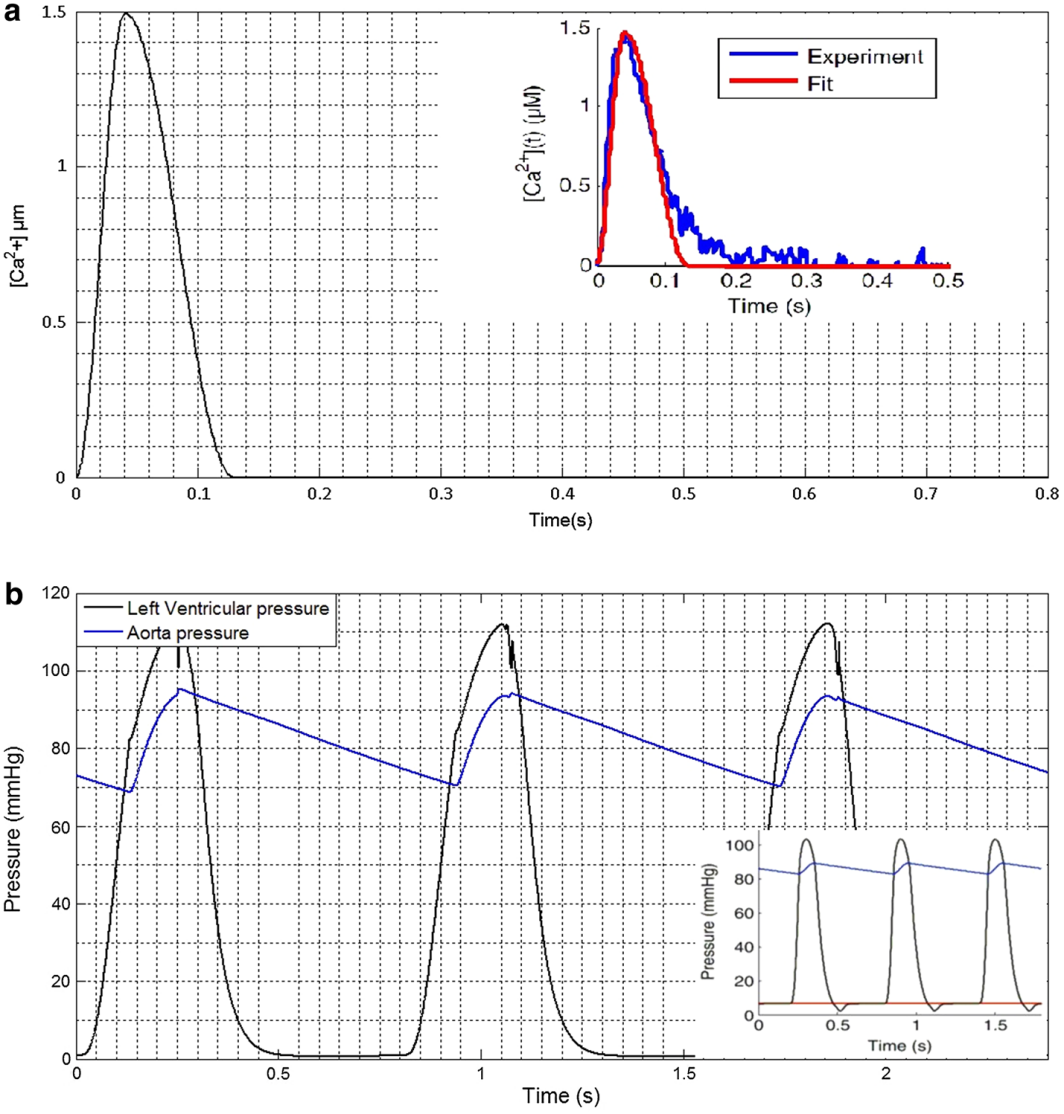
The curve of intracellular calcium transient, left ventricular pressure and aorta pressure.** a** The curve of intracellular calcium transient;** b** the left ventricular pressure and aorta pressure

To verify the validity of this model, intracellular calcium transient, left ventricular pressure and aorta pressure are simulated. According to the reference 12, values of the model parameters set up and the time of cardiac cycle is 0.8 s. The results show that the curve of intracellular calcium transient is coincident with the curve of experiment (blue curve) and fit (red curve), which originates from reference. The top concentration is 1.5 μM and the time of maximum calcium concentration is 40 ms (Fig. 2a).

The left ventricular pressure and aorta pressure are shown in Fig. 2b. From the results, we found that the tendency of pressure is similarity with reference (the curve of lower right corner, black one is left ventricular and blue one is aorta pressure). The maximum pressure of left ventricular is 110 mmHg. The systolic pressure and diastolic pressure are 95 mmHg and 70 mmHg, respectively. Based on the results, the model can be utilized to reflect and perform the hemodynamic characteristics.

To investigate the hemodynamic effects of support level and model of LVAD and LVAD coupling β-blocker on the cardiovascular system, numerical simulations were carried out. The top concentration of intracellular calcium ion is 0.54 μM [17] and 0.8 μM for heart failure and LVAD coupling β-blocker.

### Effect of support level of LVAD on cardiovascular hemodynamic

**Fig. 3:**
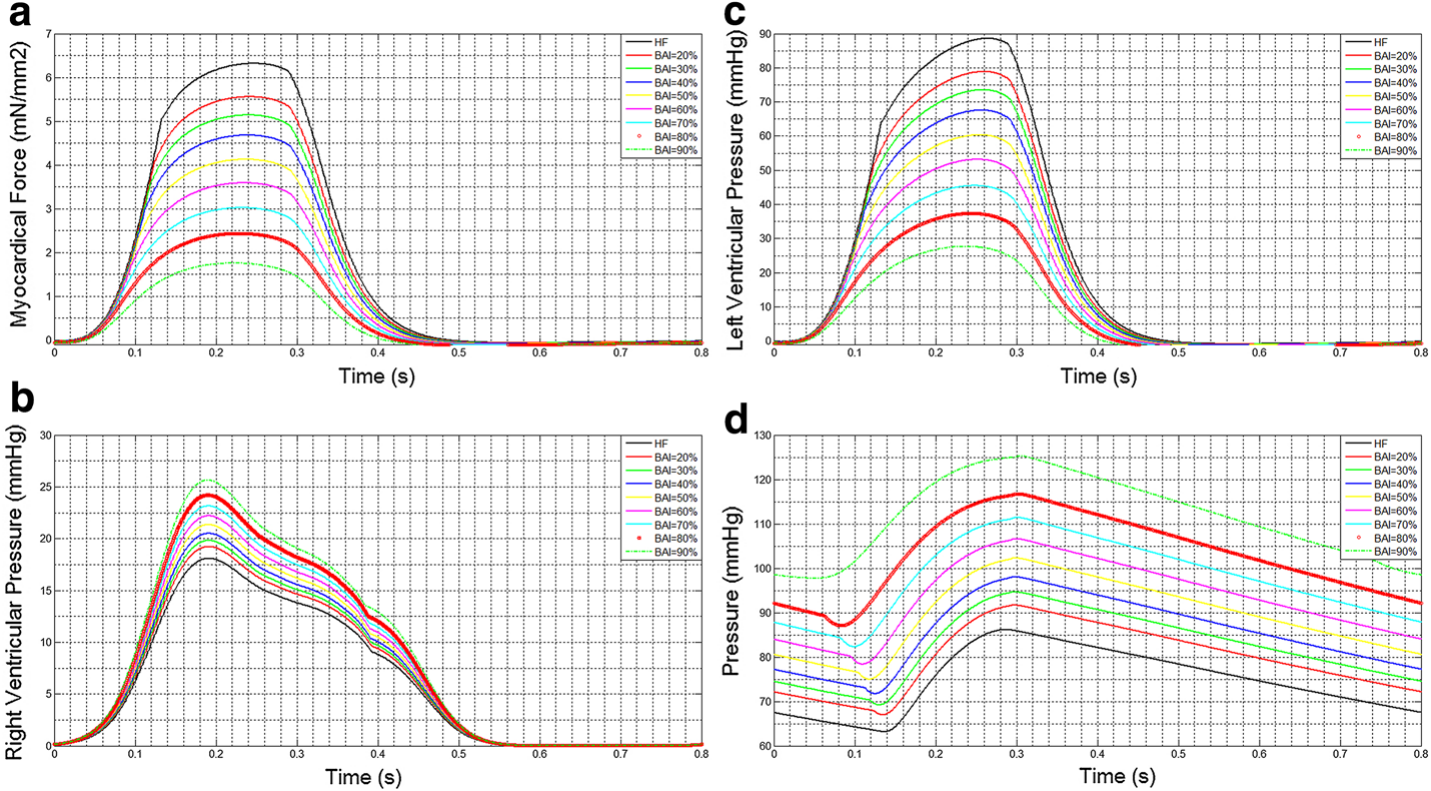
The change of hemodynamics parameters along with BAI.** a** The change of myocardial force with BAI;** b** The change of right ventricular pressure with BAI;** c** The change of left ventricular pressure with BAI;** d** The change of arterial pressure with BAI

Figure 3 illustrates the change of hemodynamics parameters along with the variation of BAI. Maximum myocardial force is found in HF patient (Fig. 3a). However, the force has the decreasing trend as the trend of the BAI increasing from 20 to 90%. Meanwhile, the peak values of LVP as support level increasing represented the unloading state of left ventricular in Fig. 3b. By contrast, RVP grows with BAI in Fig. 3c, which indicates the increased loading of right ventricular under the support of LVAD. Figure 3d demonstrates the changes of AP. It can be seen that AP increases with BAI.

**Fig. 4:**
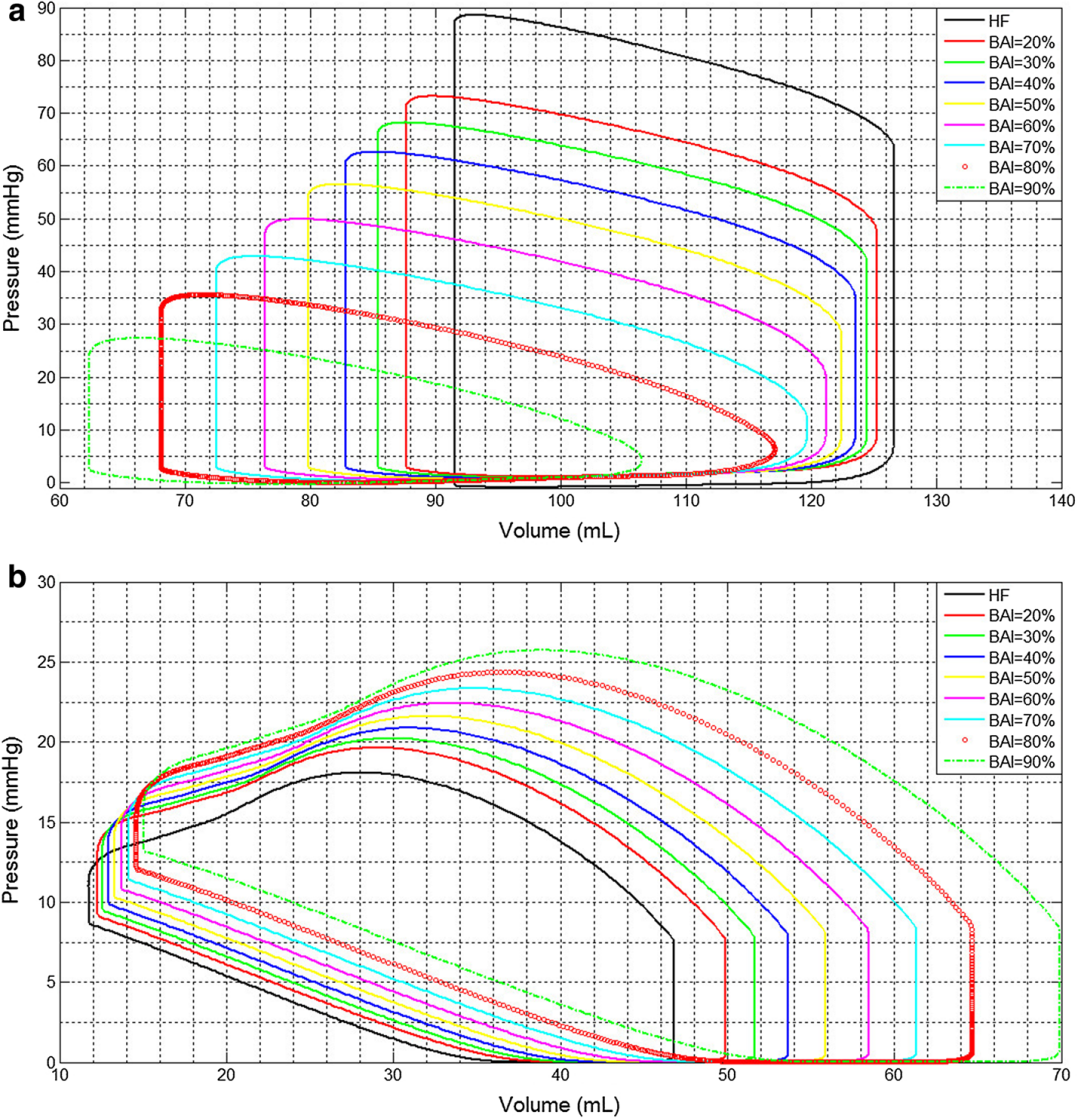
The curve of pressure–volume loop. **a** Left ventricular PV loop with support level (BAI); **b** right ventricular PV loop with support level (BAI)

Figure 4 demonstrates the pressure–volume loop. The PV loop moves toward the bottom left as BAI increase, which indicates the unloading of LV along with support level in Fig. 4a. By contrast, PV loops of right ventricular (RV) are gradually extended and moved to the right in Fig. 4b. This phenomenon indicates that RV pressure was increased the loading of LVAD. Higher support level has more effect for RV, which could increase the risk of RV failure.

**Fig. 5:**
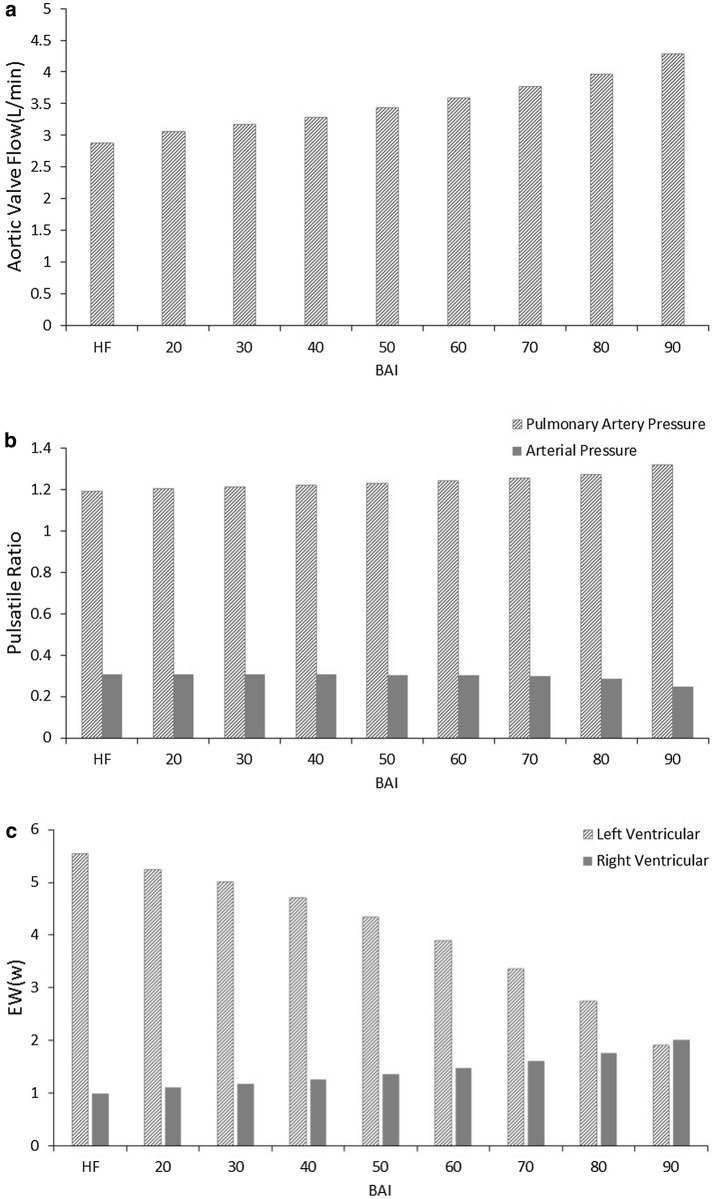
The mean flow of aortic valve, pulsatile ratio and external work. **a** The mean flow of aortic valve with support level (BAI) of LVAD; **b** PR of pulmonary artery pressure and arterial pressure with support level (BAI); **c**: EW of left ventricular and right ventricular with support level (BAI)

Figure 5 represents the mean flow of aortic valve, pulsatile ratio and external work. The mean flow of aortic valve (AV) increases steadily in Fig. 5a, ranging from less 3 L/min to more than 4 L/min (HF: 2.87 L/min; BAI 20%: 3.07 L/min; BAI 30%: 3.17 L/min; BAI 40%: 3.29 L/min; BAI 50%: 3.44 L/min; BAI 60%: 3.59 L/min; BAI 70%: 3.76 L/min; BAI 80%: 3.96 L/min; BAI 90%: 4.29 L/min), which increase the blood perfusion to organs. From Fig. 5b, it can be seen that PR of aortic pressure was reduced, and PR of pulmonary artery pressure was increased along with the growth of BAI. In addition, EW of left ventricular diminished gradually whereas EW of right ventricular gradually increased (Fig. 5c), which indicated that loading was reduced for left ventricular and increased for right under support of BJUT-II VAD.

### Effect of support model of LVAD on cardiovascular hemodynamic

**Fig. 6:**
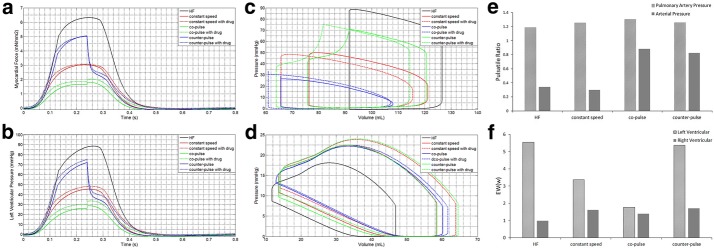
The effect of support model of LVAD on cardiovascular hemodynamic. **a**, **b** The curve of myocardical force and left ventricular pressure with support model of LVAD and LVAD coupling β-blocker at the same perfusion pressure; **c** and **d** left ventricular and right ventricular PV loop with support model and LVAD coupling β-blocker at the same perfusion pressure; **e** PR of pulmonary artery pressure and arterial pressure with constant speed, co-pulse and counter-pulse of LVAD at the same perfusion pressure; **f** EW of left ventricular and right ventricular with constant speed, co-pulse and counter-pulse of LVAD at the same perfusion pressure

To research the effect of support model of LVAD on cardiovascular hemodynamic, three support models (constant speed, co-pulse and counter-pulse) were chosen at the same perfusion pressure, which is 98 mmHg of the arterial pressure (AP). Figure 6a and b are the relationship between myocardial force and left ventricular pressure. We can find that co-pulse model has the lowest force and LVP, which illustrates the most degree of unloading. In addition, PV loops of LV and RV have the same result. PV loop of LV was significant diminution with co-pulse model than constant speed and counter-pulse model in Fig. 6c. PV loop of RV changed minimally moved toward right with co-pulse model (Fig. 6d) among the three models.

For PR of pulmonary artery pressure () and arterial pressure, co-pulse model has the maximum value of PR in Fig. 6e. In other words, co-pulse model can maintain pulsatile, which is benefit to vascular characteristic. In addition, the effect of support model on PR was more pronounced in arterial pressure than pulmonary artery pressure.

The different of EW was prominent during three models in Fig. 6f. The value of left ventricular EW was lowest with co-pulse model (HF: 6 w; constant speed: 3 w; co-pulse: 2 w; counter-pulse: 5 w), while EW of the right ventricular kept same. In conclusion, co-pulse model was the most benefit to unloading left ventricular and maintain pulsatile of pressure. Moreover, right ventricular has minimal impact by support of co-pulse model.

### Mechanism of LVAD coupling β-blocker

Above mentioned support level and model of LVAD, β-blocker as the mainstay drug for clinical treatment was researched the treatment effectiveness of LVAD coupling β-blocker. Then, the effect of β-blocker with LVAD on cardiovascular hemodynamic characteristics has been studied in this paper. Figure 6a and b also represents the curve of myocardial force and left ventricular pressure with LVAD and LVAD coupling β-blocker. Myocardial force and left ventricular pressure were lowest under co-pulse model. Compared between LVAD and LVAD coupling β-blocker, myocardial force and left ventricular pressure have no significant difference. Addition of β-blocker only has slight effect on myocardial force and left ventricular pressure.

**Fig. 7:**
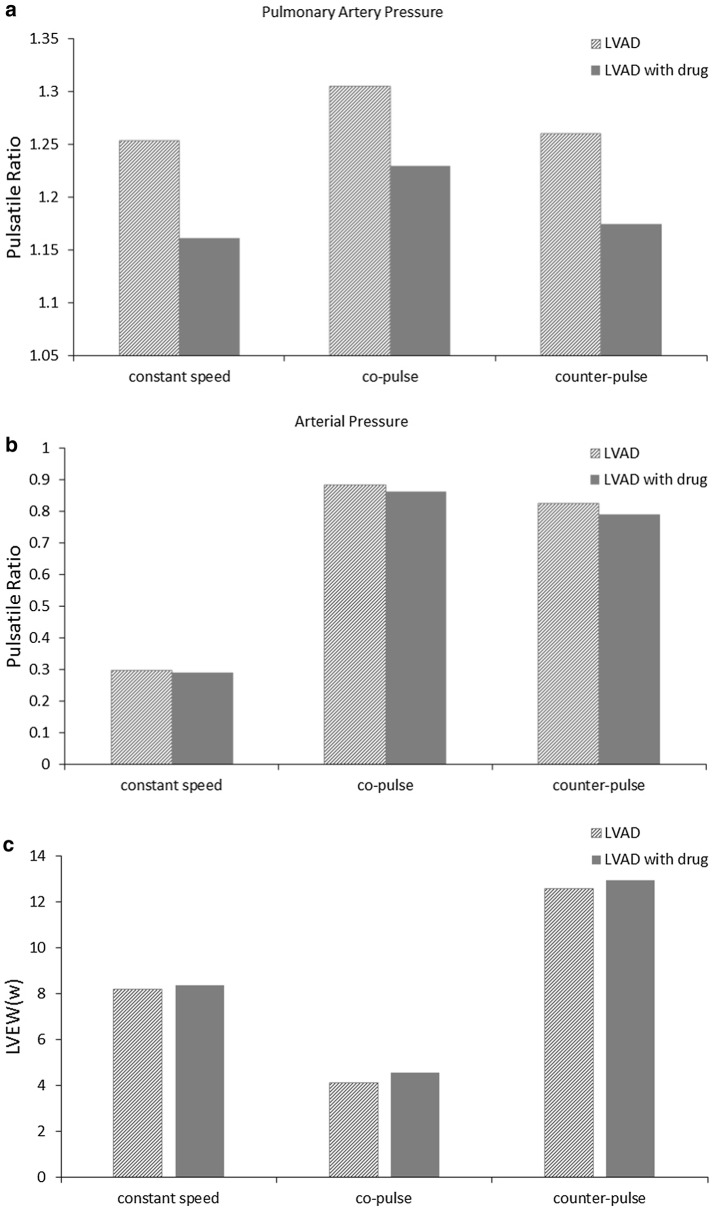
The mean pulsatile ratio and external work. **a** PR of pulmonary artery pressure with constant speed, co-pulse and counter-pulse of LVAD and LVAD with β-blocker; **b** PR of arterial pressure with constant speed, co-pulse and counter-pulse of LVAD and LVAD with β-blocker; **c** EW of left ventricular with constant speed, co-pulse and counter-pulse of LVAD and LVAD with β-blocker

Figure 6c and d were left ventricular PV loop under constant speed, co-pulse and counter-pulse for LVAD and LVAD with β-blocker. PV loop moved left under three model of LVAD with β-blocker, indicating that β-blocker was beneficial for unloading left ventricular. In addition, β-blocker reduced PR of pulmonary artery pressure (LVAD vs. LVAD with drug: constant speed 1.25 vs. 1.16; co-pulse 1.31 vs. 1.22; counter-pulse 1.26 vs. 1.17) but minimally impacted PR of arterial pressure (LVAD vs. LVAD with drug: constant speed 0.30 vs. 0.29; co-pulse 0.89 vs. 0.86; counter-pulse 0.82 vs. 0.79) in Fig. 7a and b. Similarly, β-blocker increased EW of left ventricular in Fig. 7c (LVAD vs. LVAD with drug: constant speed 8.16 vs. 8.35; co-pulse 4.08 vs. 4.55; counter-pulse 12.54 vs. 12.95). In conclusion, β-blocker achieves the dual effect of unloading and volume reduction with LVAD.

## Discussion

It has been reported the phenomenon of reverse remodeling, less extensive fibrosis, and a decrease in myocyte size have been appeared after LVAD support. However, a gap still exists between the positive morphologic changes seen and complete recovery in function. This Only 5% patients fully recovered and are eligible to accept LVAD explantation [18]. Currently, clinical studies confirmed that the LVAD with drug strategy could significantly promote the reverse cardiac remodeling [19]. However, the precise effect of LVAD and drug strategies on the cardiovascular hemodynamic states is still being studied.

This is the first study on the hemodynamic effect of LVAD with β-blocker on the cardiovascular system. In the present study, the hemodynamic effect of LVAD with or without β-blocker on the cardiovascular system has been reviewed by using a multi-scale cardiovascular model. The results demonstrated as support level of LVAD increased, the pressure and external work of the left ventricle were gradually reduced, while the pressure and external work of right ventricular increased. When the LVAD support was combined with β-blocker, the left ventricular volume was reduced significantly. However, the pressure and external work of left ventricle did not have any difference between LVAD β-blocker couple and only LVAD. Myocardial force has been considered as a very critical factor related to the myocardial remodeling [20]. Di Napoli et al. [21] firstly reported that the myocardial force is a direct correlate of cardiomyocyte apoptosis in patients with severe dilated cardiomyopathy. Choi et al. [22] proposed that myocardial force could regulate the myocyte apoptosis. According to these reports, increased myocardial force attracted more attention due to its correlations on cardiac structure and function. Krittayaphong et al. [23] found correlations between left ventricular wall stress and N-terminal pro-brain natriuretic peptide (NT-pro-BNP) levels. Nishimura et al. [24] proposed that a sustained increasing in myocardial force was likely to elicit eccentric or concentric left ventricular remodeling.

Myocardial force overload, even at its earliest stages, is not well tolerated by the developing ventricle and myocardial force affected the cardiac function. Myocardial force is crucial which is closely related to regional coronary blood flow [25], myocardial oxygen consumption [26], hypertrophy [27], and cardiac molecular systems connected to the development of long-term cardiac insufficiency [28]. LV remodeling may be suggested by the significantly reduced end-systolic myocardial force observed for patients with pericardial effusion when compared to healthy control patients. Therefore, force is believed to be responsible for adverse cardiac remodeling [29]. In LVAD applications, multiple studies found that chronic LVAD support reduces force, and this may contribute to reverse remodeling [30, 31]. Thus, the application of LVAD has positive results. LVAD is found to reduce myocardial force by pumping blood out of the left ventricle. Jhun et al. [32] investigated the changes in myocardial force under LVAD support by using computational fluid dynamic analysis (CFD). He found that the myocardial force under LVAD support was significantly reduced. Our study results are consistent with the mentioned above studies. The myocardial force (Fig. 3a) under LVAD support was decreased along with the increase of support level of LVAD. The results demonstrated that LVAD support could reduce myocardial force and then provide an optimal mechanical environment for promoting cardiac function. In addition, results demonstrated that the support modes of LVAD have significant effects on the myocardial force (Fig. 6a). Myocardial force under co-pulse mode exhibited the lowest net pressure amongst the rest, suggesting that co-pulse support mode could achieve the greatest left ventricular unloading performance. Addition of β-blocker to LVAD did not produce significant changes in myocardial pressure. This indicates that β-blocker, alone, likely does not have any effects on promoting reverse cardiac remodeling [33], emphasizing that much of unloading is due to LVAD. Nevertheless, the combination of LVAD and β-blocker has attracted more and more interest due to its exciting effects on improving cardiac function [19, 34]. However, the mechanism and pathway of β-blocker in the therapy is still unclear. In general, when β-blocker being combined with LVAD, left ventricular volume was significantly reduced compared with that of only LVAD support, indicating an ability to affect cardiac tissue. However, the left ventricular pressure under both cases does not have any significant differences. That means the β-blocker, used in this study, mainly played a role in reduce the left ventricular volume. This is the first study that proposed this phenomenon, which maybe provide a new insight to the combination of LVAD and β-blocker. In this paper, Ca^2+^ released model and cross-bridge dynamics model were used to calculate the myocardial force. Some fine structures, attached to the ventricular wall, have been ignored. However, these structures are very important in understanding the functionality of human heart and in the diagnostic of cardiac diseases. Reconstruction the trabeculae through topological prior method proposed by Wu to study the stress of ventricular wall [55]. In the future, we will consider these structures in our model.

The myocardial overload has been considered as an exacerbation, leading to damage of myocardial remodeling, myocardial structure and function [35]. The two kinds of overload are pressure overload and volume overload [36]. The pressure overload, caused by the abnormal increase of cardiac afterload (valvular stenosis or aortic stiffness), leads to the concentric myocardial hypertrophy. Cardiac ventricular volumes have been widely used as a measurement of cardiac abnormalities and functions [54]. The volume overload, caused by the abnormal increase in left ventricular preload, leads to the eccentric myocardial hypertrophy. Toischer et al. [37] found that the pressure overload results in maladaptive fibrotic hypertrophy with CaMKII-dependent altered calcium cycling and apoptosis, and the volume overload was associated with Akt activation without fibrosis. Zhen et al. [54] propose direct and simultaneous four-chamber volume estimation showing high performance both MR and CT images. Studies demonstrated that the continuous flow LVAD (CF-LVAD) achieves pressure unloading, while the pulsatile flow LVAD (PF-LVAD) mainly achieved volume unloading [30]. A clinical study found that the patient implanted PF-LVAD was more likely to have reverse cardiac remodeling than that supported by CF-LVAD [38]. It appears pulsatile volume unloading that may be more important than pressure unloading. Prior researches suggest that reduced pulsatility may contribute to ischemic and hemorrhagic stroke [39] and oxidative stress [40], as well as increased aortic stiffness [41]. Therefore, many studies improving volume unloading performance focus on designing pulsatile control strategies for CF-LVAD [42]. The present study demonstrated that the left ventricular volume had been significantly reduced under LVAD combined with β-blocker, compared with that under only LVAD support (Fig. 6c and d). In contrast, the left ventricular pressure didn’t much change with the addition of β-blocker. That means the β-blocker could significantly improve the volume unloading performance of CF-LVAD, without affecting pressure unloading performance of CF-LVAD. Thus, LVAD combined with β-blocker could significantly promote reverse cardiac remodeling. When β-blocker is combined with co-pulse mode, our model suggests the best volume unloading and pressure unloading performances. This means the co-pulse mode with β-blocker could an optimal strategy for promoting cardiac structure and function recovery and would be a nice direction for future study.

VEW was used to evaluate the declining cardiac performance in heart failure patients [43]. Model showed that EW of left ventricle was significantly affected by the support level and support modes of LVAD. The left ventricular EW was decreased as support level of LVAD increases (Fig. 5c). In addition, among the three support modes, the co-pulse mode achieved the lowest left ventricular EW and the counter-pulse mode achieved the highest left ventricular EW (Fig. 6f). The system added β-blocker did not affect left ventricular EW. This phenomenon is benefit for designing the combined strategy of LVAD and β-blocker. According to current studies, the excess left ventricular EW could impair the cardiac structure and function [44]. Hence, the left ventricular EW regulated precisely is important and many strategies have been proposed toward this goal. If adding β-blocker into LVAD does not affect left ventricular EW, previously established LVAD strategy to reduce volume is possible. This characteristic has benefit for promoting the application of LVAD combined with β-blocker in clinical practices.

Currently, the effects of LVAD support on the right ventricular function have been paid increasing attentions. Many studies found that the patients, supported by LVAD, have high probability of suffering from right heart failure [45]. For instance, Gupta et al. [46]. reported that the changes in left ventricular hemodynamic states after LVAD implantation were the key causes for the occurrence of right heart failure. Argiriou et al. [47] indicated that the abnormal preload of right ventricular was also an important factor. The present study shows the right ventricular EW, supported only by LVAD, was increased with the growth of support level of LVADs. In other words, the load of the right ventricular was increased by the LVAD support (Fig. 5c). Moreover, model results demonstrated that the support modes also change right ventricular EW (Fig. 6f). LVAD under co-pulse mode achieved lowest right ventricular EW among the three support modes. However, it is still higher than that without LVAD support. That means LVAD support increase the right ventricular EW, which may be a reason for the occurrence of right heart failure. This phenomenon is good for improving the design of optimal support mode to regulate the right ventricular EW to prevent heart failure.

In this study, a lumped parameter model was utilized to clarify the interaction between cardiovascular system, LVAD and β-blocker. Lumped parameter model has been widely used to study the hemodynamic states of cardiovascular system under both healthy and pathological conditions [48–50]. These studies demonstrated that the lumped parameter model could accurately reproduce the hemodynamic states of cardiovascular system under various condition. In addition, finite element method is used to study the cardiovascular system through CT/MRI data. Recently, deep temporal regression network [51] and state-space framework [52] have been reported to recognize the MRI frames or ultrasound sequences, which can improve the accuracy. Zhang et al. [53] proposed meshfree method, which can conveniently process the numerical computation inside interested domains with large deformation or inhomogeneity. In the future, the animal experiment will be conducted to study the interaction of cardiovascular system, LVAD support and drug therapy.

## Conclusions

In this study, the effect of support level and model of LVAD on cardiovascular hemodynamic characteristics is investigated and the effect of β-blocker on unloading with LVAD is analyzed to elucidate the mechanism of LVAD coupling β-blocker. A multi-scale model including calcium transient of human ventricular myocyte, the cross-bridge dynamics model, ventricle model and cardiovascular-pump system model was established. It is used to study hemodynamics of cardiovascular system with LVAD coupling β-blocker. Myocardial force, AOP, LVP, AP, aortic valve flow, PV loop, EW and pulsatile ratio represented state of the cardiovascular system. The results demonstrate that increase of support level of LVAD, the pressure and EW of the left ventricle decreased, but increased for right ventricular. β-blocker added into LVAD did not change pressure and EW of left ventricular. However, β-blocker led to significant reduced to left ventricle volume. Then, there is a suggesting that it can be a therapeutic for reducing cardiac remodeling when using LVAD.
